# Experiences from Cyclone Anna and Cyclone Dumako: A short report

**DOI:** 10.4102/phcfm.v14i1.3761

**Published:** 2022-12-14

**Authors:** Amos Mailosi, Stanley Mwalwanda, Charles Hassan, Stalin Zinkanda, Beatrice Matanje, Fabien Munyaneza, Moses B. Aron, Emily Dally, Manuel Mulwafu, Chiyembekezo Kachimanga

**Affiliations:** 1 Department of Family Medicine, School of Medicine and Oral Health, Kamuzu University of Health Sciences, Blantyre, Malawi; 2 District Health and Social Services, Chikwawa District Council, Chikwawa, Malawi; 3 Partners in Health, Neno, Malawi; 4 Partners in Health, Boston, United States of America

**Keywords:** Cyclone Ana, Partners In Health, primary health care, family medicine, continuation of care, climate change, Malawi

## Abstract

In the third week of January 2022, the southern districts of Malawi were hit by Cyclone Ana. The worst affected areas were Chikwawa and Nsanje. Four weeks following Cyclone Ana, a rather smaller cyclone, Dumako, hit the same areas, causing more damage. The Partners in Health or Abwenzi Pa Za Umoyo, an international humanitarian nongovernmental organisation that provides primary health care (PHC), organised teams to join Chikwawa District Council – Health, providing PHC assistance in the most affected district (Chikwawa); these teams were joined by three senior residents in family medicine from Kamuzu University of Health Sciences.

**Contribution:**

From the experiences of the interventions reported here, it was learnt that a multidisciplinary team of PHC providers is the key to the success of the emergency PHC programmes in times of natural disasters. While immediate PHC may be important at the actual time of disaster, it was learnt that PHC is also very important for continuation of care for chronic conditions, antenatal clinics and other clinics that are interrupted by the disaster. The experiences emphasised the importance of involving the PHC physicians and other PHC cadres in planning PHC programmes in natural disaster–prone areas.

## The context

Recently, Malawi has been hit by several natural disasters, particularly severe weather-related floods, exacerbated by global climate change.^1^ The southern region of Malawi, Chikwawa and Nsanje districts, are the most prone to flooding because of their low-lying positions, as they are below sea level. The districts lie in a valley where the Shire River, which is the outlet of Lake Malawi to the Zambezi River, cuts across these two districts. During the rainy season, there is usually some degree of flooding as any significant rise in the water level in Lake Malawi spills over. Despite these yearly floods, about 900 000 people live in the two districts, and a good number settle near the riverbanks because of the productivity of the land.^2^

When Cyclone Ana hit, a total of 46 people died, and 206 were injured across the country; 21 of the 46 deaths were in Chikwawa district (CD). An estimated 990 000 people needed urgent medical and humanitarian support, including emergency shelter for approximately 190 000 displaced persons.^3^ Chikwawa district was the hardest hit, with thousands of people living in 92 camps. Some of the health facilities were destroyed, resulting in the unavailability of essential health services.

## Provision of health services in camps

At the request of CD Council in conjunction with Ministry of Health (MoH), Partners in Health (PIH) Malawi, known locally as Abwenzi Pa Za Umoyo (APZU), in collaboration with the Family Medicine (FM) Department of Kamuzu University of Health Sciences (KUHeS), supported CD with the provision of medical care to people living in camps as an immediate response to the disaster. Partners in Health Malawi is a nongovernmental organisation (NGO) that has worked in another district in Malawi for over 15 years to support the MoH in the provision of primary health care (PHC). Due to the dire need for support, PIH decided to provide urgent support to the population of Chikwawa.

The team worked with the Chikwawa District Health Office (DHO) to: (1) strengthen the district’s coordination of partners working in the provision of health services; (2) provide essential health services to people living in camps; (3) support the supply of medication, human resources and other logistics needed for the provision of medical care; and (4) support the collection of health information during the mobile camp visits.

The team supported the district to coordinate the weekly health cluster meetings where the DHO and all partners discussed the overall health response to the disaster. Guided by the DHO, the PIH team supported the initial and follow-up health cluster meetings. The overall health cluster response plan was developed.

To initiate the camp visits, five mobile teams were identified. The composition of the team members was guided by the package of care that would be provided to the camps. The package of care included:

1. Provision of acute healthcare services. Patients who were acutely ill received care. Partners in Health coordinated care for patients who required secondary level care by providing referral services from the camps to the district hospitals. These referral services included the decision to refer the patients and also provision and facilitation of transportation.
2. Continuation of chronic disease care pathways. The researchers of this study ensured that patients living with human immunodeficiency virus (HIV) and noncommunicable diseases (NCDs) had adequate medication and supplies and they were enrolled in chronic care clinics.
3. Strengthening the provision of care for reproductive and maternal health services. Family planning was offered, as well as antenatal care (ANC), postnatal care, sexually transmitted infection (STI) services and malnutrition screening and treatment to the people living in the camps.
4. Preventive health services. Camp committees were educated to provide education on water and sanitation and communicable diseases such as coronavirus disease 2019 (COVID-19), as well as distributing mosquito nets.
5. Common mental disorders were screened for and provided mental healthcare to the mental health clients, such as people with depression and anxiety disorders and victims of sexual and gender-based violence (SGBV), among others.
6. Screening was included and care for SGBV, as these are common when people are displaced from their homes.
7. A simple formulary of medications needed to cover the priority conditions in the camps were created. Because of shortages of medication, extra medication and supplies were procured and donated to the main district hospital for use in the camps.

Each team was composed of one to two medical doctors, clinical officers or technicians, a pharmacist or pharmacy technician, two to three nurses, an environmental health assistant, a laboratory technician, an HIV diagnostic assistant and a social worker. Camps that needed to be visited were identified by the Chikwawa DHO, starting with extremely hard-to-reach camps and aiming to visit each camp at least once a month. Partners in Health provided medical supplies and human resources (two doctors, two nurses and two pharmacy personnel every week), with the rest of the staff provided by the MoH. Partners in Health also provided two vehicles and other logistics for the camps.

## The role of family medicine residents or doctors and other primary health care physicians

At the time, the district had only two doctors available. Care at the primary care level is mainly provided by clinical officers (usually with a 3-year clinical training), medical assistants (2-year college training) and nurses. Malawi has a well-structured PHC system, but a poor distribution of resources leaves the rural areas like Chikwawa with an inadequate workforce, even when there is no disaster.^4^ The FM residents, together with PIH physicians, clinicians, nurses and other cadres, provided PHC to the victims. The residents were included because: (1) it was an opportunity for them to be involved in disaster management, as part of their training; (2) they were better placed in the provision of PHC than residents from other specialities. There is a good relationship between PIH and FM, as PIH focuses on providing high-quality PHC in Malawi. Additionally, residents also benefitted by learning how to plan and implement an intervention in PHC in times of disaster.

Overall, the interventions benefitted the flood victims through the provision of PHC. Under the usual circumstances, less than 50% of Chikwawa’s population lives within the 5 km radius of health facilities recommended by the World Health Organization (WHO). This implies that even in the absence of a disaster, PHC is not accessible to the people living in the hard-to-reach areas.

## The outcome

**FIGURE 1:**
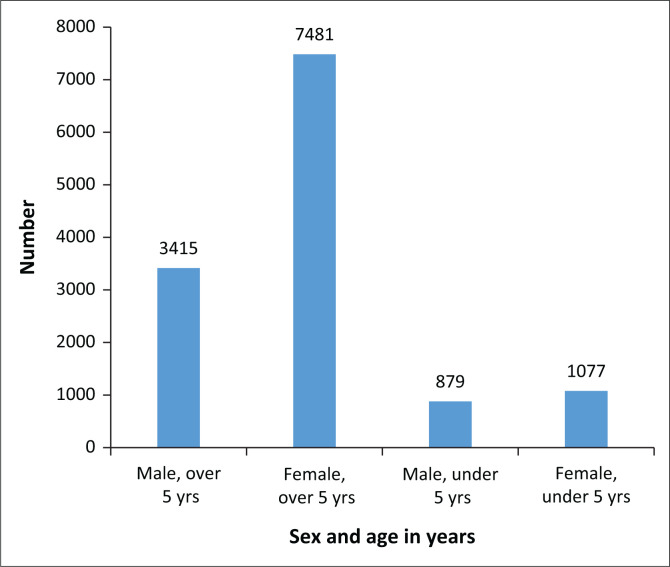
Utilisation of primary health care services in Partners in Health–supported camps.

Between January 2022 and April 2022, the Chikwawa DHO conducted 277 mobile clinic visits to 71 camps. Among the mobile clinic visits, 41% were directly supported by PIH. Figure 1 shows the utilisation of services in PIH-supported camps. This is in agreement with a previous study that shows higher utilisation of services by female patients in comparison to male patients.^5^ The syndromic management approach was used to diagnose and treat STIs without laboratory tests. This requires competent physicians who can work with limited resources.

**FIGURE 2:**
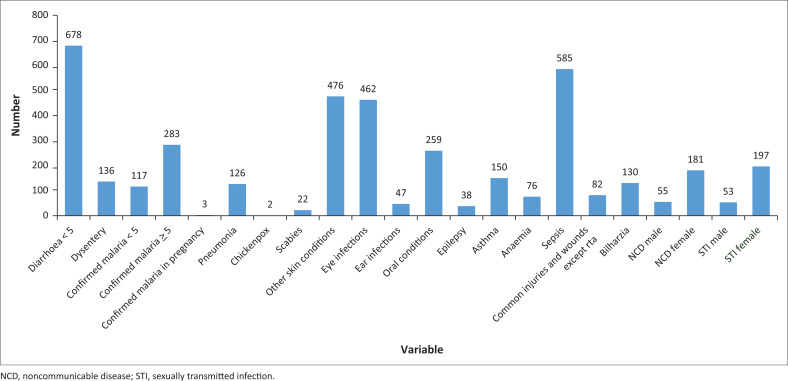
Diagnoses in people attending primary health care in Partners in Health – supported camps.

Diarrhoea was the most common condition, followed by skin conditions and sepsis (Figure 2). The majority of the diarrhoeal conditions were among patients under 5 years. The causative organisms for diarrhoea could not be identified but based on epidemiological data in Malawi, and suspect viral causes such as the rotavirus.^6^

While sepsis is a medical emergency, most of the cases reported may not have been sepsis. In this setting, where it was not possible to laboratory investigations to support the diagnosis, a good history, physical examination and a high suspicion for bacteria as a possible cause of the symptoms were enough to treat for suspected sepsis or bacteraemia with antibiotics.

**FIGURE 3:**
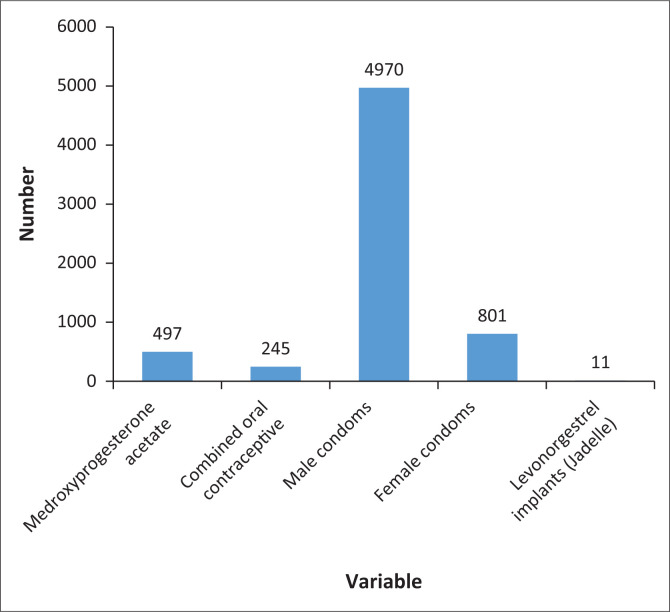
Family planning methods distributed in the camps.

Figure 3 summarises the contraceptives that were distributed in the camps during the mobile visits. The researchers of this study mainly distributed condoms, followed by depot medroxyprogesterone acetate, combined oral contraceptives (progestin and oestrogen) and levonorgestrel implants (Jadelle implants).

**FIGURE 4:**
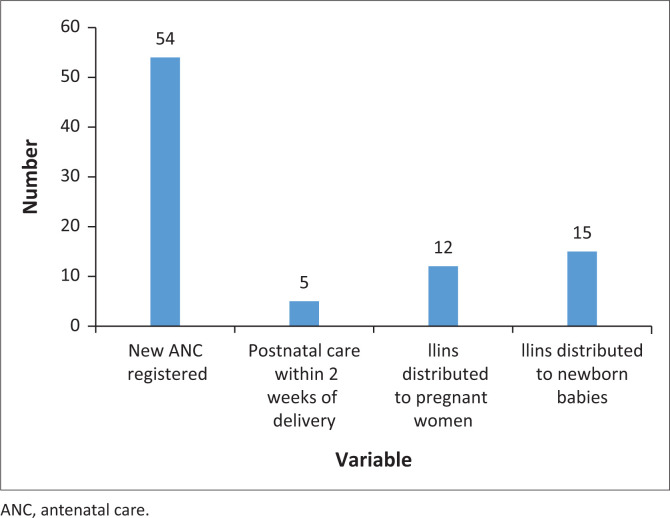
Antenatal clinic and postnatal care services at the camps.

Among women of reproductive age group, 54 women registered for their first ANC visit, of whom 36 registered in first trimester (see Figure 4). Previous studies have reported that over 95% of pregnant women in Malawi attend at least one antenatal visit.^7,8^ Malawian women have good knowledge about starting ANC in time, but they may prefer to have fewer visits or to start later because of social factors.^9^

**FIGURE 5:**
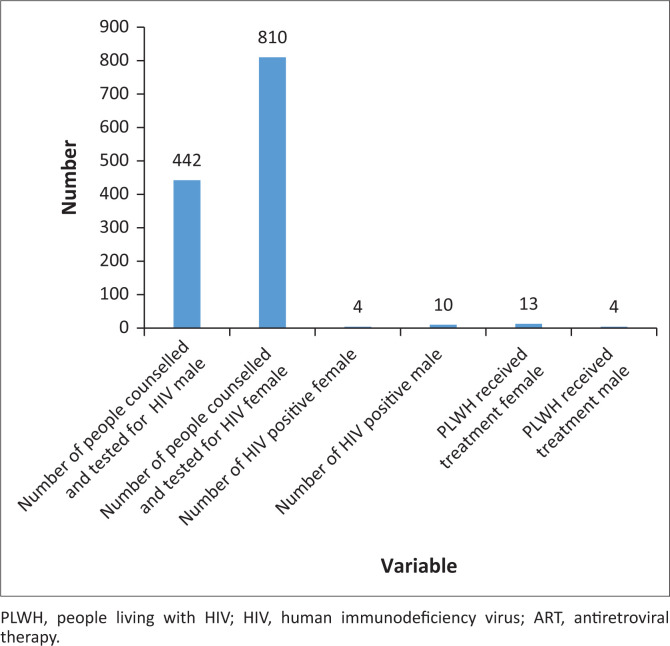
HIV and ART services.

Figure 5 summarises the HIV-related services. The researchers of this study tested 1252 clients for HIV and found 14 (1.1%) who were HIV positive. The clients who tested HIV positive were counselled, initiated on treatment and linked to a health facility in their area.

## Lessons learned

Several lessons were learnt during the emergency response, including the need to support the coordination of care at the district level, the comprehensiveness of care packages including SGBV, mental health and the need for rapid mobilisation of resources to support care in the camps. Primary health care was provided for 5 weeks; and left when all the health facilities were fully open and functional. The PHC provision to tropical cyclone victims was urgently needed for people living in the camps. The intervention ensured the continuation of care to patients with chronic conditions and also the provision of acute care for patients with various conditions. The access to PHC for many people who would otherwise have no easy access to healthcare was improved. Good examples are the 54 women who attended ANC for the first time and the 14 new HIV clients who were started on treatment.

The authors noticed challenges with the provision of mental health services. They did not have a specialised team to provide such care; there were no psychosocial counsellors or mental health clinicians. While PHC physicians can provide primary mental healthcare, ongoing care from mental health clinicians or psychosocial counsellors would be ideal to provide more comprehensive mental care. Additionally, the focus were on the provision of essential care in camps but did not address the infrastructure problems and provision of care at the PHC facilities that were still functioning.

This experience is crucial to future planning for interventions in times of natural disasters that have now become more prevalent because of unpredictable global climate changes. The experience also shows the crucial role that PHC providers play. The role of FM residents and physicians in the provision of emergency PHC cannot be over-emphasised.

The authors recommend that FM residents and physicians should be involved more in planning for emergency PHC. They recommend having a mental health team involved in providing PHC to internally displaced victims living in camps. They also acknowledge that the need for interventions by NGOs, such as PIH, in collaboration with the MoH, may be recurrent and need to persist for longer as areas take many months to recover from the effects of natural disasters.

Partners in Health (PIH) is acknowledged for funding the interventions reported in this report, as well as the publication of this report. The authors also acknowledge the Department of Family Medicine at Kamuzu University of Health Sciences for allowing the family medicine registrars time to be part of this project. There are many names from Chikwawa District Council with whom the authors worked side by side to assist the cyclone victims; their memories of these people strengthened our inspiration and motivation to write this report.

## References

[1] Bahadur D, Prakash J, Marenya P. Understanding climate-risk coping strategies among farm households: Evidence from five countries in Eastern and Southern Africa. Sci Total Environ. 2021;769:1–19. 10.1016/j.scitotenv.2021.145236 33736234

[2] Government of Malawi: National Statistical Office. 2018 Malawi population and housing main report. 2019; p. 1–299. Available from: https://malawi.unfpa.org/sites/default/files/resource-pdf/2018%20Malawi%20Population%20and%20Housing%20Census%20Main%20Report%20%281%29.pdf

[3] United Nations Malawi. Malawi - Flash Appeal - Tropical Storm Ana, February - May 2022 [homepage on the Internet]. [cited 31 July 2022]. Available from: https://malawi.un.org/en/173845-malawi-flash-appeal-tropical-storm-ana-february-may-2022

[4] Makwero M. Delivery of primary health care in Malawi. Afr J Prim Health Care Fam Med. 2018;10(1):a1799. 10.4102/phcfm.v10i1.1799 PMC6018651 29943590

[5] Kasenda, S., Meland, E., Hetlevik, Ø. et al. Factors associated with self-rated health in primary care in the South-Western health zone of Malawi. BMC Prim. Care 23, 88 (2022). https://bmcprimcare.biomedcentral.com/articles/10.1186/s12875-022-01686-y#citeas 35439944 10.1186/s12875-022-01686-y PMC9016970

[6] Clark A, Black R, Tate J, et al. Estimating global, regional and national rotavirus deaths in children aged <5 years: Current approaches, new analyses and proposed improvements. PLoS One. 2017;12(9):e018. 10.1371/journal.pone.0183392 PMC5593200 28892480

[7] Pell C, Meñaca A, Were F, et al. Factors affecting antenatal care attendance: Results from qualitative studies in Ghana, Kenya and Malawi. PLoS One. 2013;8(1):e53747. 10.1371/journal.pone.0053747 23335973 PMC3546008

[8] Office NS. Demographic and health survey key indicators 2015–16_Malawi [homepage on the Internet]. 2015; p. 1–45. Available from: https://dhsprogram.com/pubs/pdf/FR319/FR319.pdf

[9] Chimatiro CS, Hajison P, Chipeta E, Muula AS. Understanding barriers preventing pregnant women from starting antenatal clinic in the first trimester of pregnancy in Ntcheu District-Malawi. Reprod Health. 2018;15:158. 10.1186/s12978-018-0605-5 30241542 PMC6151039

